# Supplementary material for: Transformation of alignment files improves performance of variant callers for long-read RNA sequencing data
Source: Genome Biol. 2023 Apr 24;24:91. doi: 10.1186/s13059-023-02923-y (PMC10123983; doi:10.1186/s13059-023-02923-y)
Supplement: Supplementary file 2 — Additional file 2. Review history. [file 13059_2023_2923_MOESM2_ESM.docx]

**1^st^ round**

**Reviewer 1**

This paper explores an aspect of variant calling on long read RNA-seq, specifically, ISOseq. Variant calling tools and BAM processing are explored along with features such as coverage and proximity to splice sites. The comparisons are performed appropriately with regard to statistical evaluations of precision, recall and F1 score.

One major concern I had were that the descriptions were very confusing and there was a lot of jargon used.

I actually think the main result of this manuscript is that you need to correct coverage for intronic sequence where no information is available for variant calls. This paper gives a tool for doing this. Unfortunately, I felt this point was poorly communicated to the extent that I'm not entirely sure if there are other results here. I apologise if I have got this wrong but I guess that is making the point that the communication of the result is very confusing. For example, the description of N-cigar-reads and proportions of N-cigar-reads in the analysis is more confusing than it needs to be. The concept here is that coverage of reads at a site cannot include the regions where reads are split and have no sequence. Why use the metric proportion of N-cigar-reads instead of calculating the "sequence coverage at a site" which is coverage/proportion(N-cigar-reads)? Is the ability to detect variants any different if there are 10 reads covering a site with 0 proportion of N-cigar-reads or if there are 20 reads and 0.5 N-cigar-reads so that the actual coverage of a site is 10? Why not have a plot that shows coverage calculated with no flagCorrection vs coverage with flagCorrection and look at the precision and recall in this context. Figure S3 is related but overall uninformative and Figure S4 is confusing because if N-cigar-proportion = 1 then there is no coverage on the variant.

Another concern I had was that I felt the scope was a little too narrow and therefore some aspects of analysis were not addressed and so left me with lingering questions about doing this analysis.

What effect does the quality of the initial alignment by minimap2 have on the variant calls? How much would a better alignment matter here?

The authors mention in the conclusions that the pipelines should work for Oxford nanopore sequencing and I think the scope would be increased substantially if this data was also included in the investigations of tool performance. There are plenty of publicly available cell line data sets that could be used for this.

Similarly, why not use well validated calls from genomic sequencing for validations as well as the complementary short read as tested already? For example, sequencing of Genome in the Bottle sample. Clearly it depends on transcript coverage but so does the RNA-seq data shown and then the coverage for truth data could be determine by the long-read data only. Are there any locations where the long read is actually better than the short read (Indels?).

**Reviewer 2**

Souza et al. benchmarked several variant callers on PacBio RNA-seq data in this manuscript. And they found that transformation of the alignment files (BAM) is critical to achieve better performance of small variant calling. The idea is interesting. I have some concerns that the authors might consider.

1. It is unclear whether the noisy long-read data has the advantage in detecting variants of RNAs compared with the short-read data. That is the key question of the work and we need a justification to do this work. The authors should compare the performance of variant calling between the short-read data of RNAs and the long-read data. What's the advantage of using long-read RNA data to call variants. As a benchmark paper, many important results were not presented in the manuscript, making it difficult to clearly understand the benchmark result or exact improvement of the performance of the variant calling.
2. It is not clear how to split the reads at intronic regions. We note that the mode of "splice" of minimap2 was used in the code submitted to github (https://github.com/vladimirsouza/lrRNAseqVariantCalling). Is this related to this purpose? Whether does a user need a gene annotation file, such as the gff (gtf) file of GRCh38. What to do if the isoform of RNA was not recorded in the annotation file? The authors need to describe how to split the reads in detail in the Methods because the accuracy of read alignment has a big impact on variant calling.
3. The accuracy of variants depends largely on the read depth, especially for the noisy long-read data. The authors should evaluate the impact of the read depth on the accuracy of the variant calling. For instance, the performance of the variant calling for the total dataset with given median coverage, and for the subsets of different coverages coupled with the site sizes in this dataset. In the end, the authors should give a recommendation on the need of sequencing depth to achieve better performance of variant calling.
4. The authors found that SNCR+flagCorrection+Clair3 had relatively poor performance to call SNPs for Clair3. And the NanoCaller failed to call variants if adding "flagCorrection". The authors claimed that the SNCR+flagCorrection could improve the performance of variant calling for GATK. However, we did not observe obvious improvement of the calling performances, at least for two tools. This seemed not to match the title of the manuscript "Transformation of alignment files improves performance …".
5. Many important definition should be well-defined. For example, In "when read coverage is low…when the coverage is high…", "low" and "high" should be clear and give values or at least a range. The same to the line 45-46 of the page 7: "most of the true variants were called".
6. The authors claimed that the NanoCaller failed to call variants from Iso-Seq data. The authors of NanoCaller claimed that the F1-score for HG002 CLR data was at least 0.93 for calling SNP. We suggested that the authors found the reason.
7. Fig. 1D, why did precision decrease as the coverage increased for calling indels using SNCR+GATK? The authors should show the change of variant number for different read coverages.
8. Fig. 1F and Fig. 2D, the thresholds of coverage were 30 and 20, respectively. Did the authors have criteria for selecting thresholds?

**Reviewer 3**

The authors show that variant calling from long read RNA-seq can generally be improved across callers when two different post-processing steps are performed on the bam: splitting reads at introns and correcting flags. They demonstrate this on 2 cell lines relative to a custom "ground truth" from illumina WGS. I expect this will be valuable for the community, but it would likely be good to add a comparison to GIAB benchmark variants, since iso-seq data are now available as described below.
1. GIAB recently made available iso-seq for its HG002 lymphoblastoid cell line (https://www.ncbi.nlm.nih.gov/sra/SRX14226557[accn], which also has illumina RNA-seq at https://www.ncbi.nlm.nih.gov/sra/SRX12200334[accn]) as well as two different iPSC lines from the same HG002 individual that have more genes expressed (https://www.ncbi.nlm.nih.gov/sra/SRX14226558[accn] and https://www.ncbi.nlm.nih.gov/sra/SRX14226556[accn]). GIAB plans to make additional short and long read RNAseq data available shortly. Importantly, variants from these could be compared to the latest GIAB benchmarks: v4.2.1 vcf and bed (which used short, linked, and long reads for the whole genome at https://doi.org/10.1016/j.xgen.2022.100128), and the 273 challenging medically relevant gene benchmark vcf and bed at https://rdcu.be/cGwVA. These benchmark variants and regions would likely provide more accurate performance metrics, since they used multiple technologies and were highly evaluated.
2. What are the n=x counts in Fig 2C? I'm surprised n is so large for mid-size homopolymers
3. In Fig 2D, I'd expect the effect of ASE to depend on both how strong the ASE is and what coverage the ASE site has in PacBio. Would the authors be able to look at either of these factors as well?
4. It also seems like it could be useful to look at the effect of coverage on variant detection at non-ASE sites

**Authors’ response**

Reviewer #1: This paper explores an aspect of variant calling on long read RNA-seq, specifically, ISOseq. Variant calling tools and BAM processing are explored along with features such as coverage and proximity to splice sites. The comparisons are performed appropriately with regard to statistical evaluations of precision, recall and F1 score.
One major concern I had were that the descriptions were very confusing and there was a lot of jargon used. I actually think the main result of this manuscript is that you need to correct coverage for intronic sequence where no information is available for variant calls. This paper gives a tool for doing this.
We thank the reviewer for this comment. We agree that many descriptions could have been clarified. We have extensively edited the manuscript, including the aforementioned section on intronic alignments, for improved clarity.

Unfortunately, I felt this point was poorly communicated to the extent that I'm not entirely sure if there are other results here. I apologise if I have got this wrong but I guess that is making the point that the communication of the result is very confusing. For example, the description of N-cigar-reads and proportions of N-cigar-reads in the analysis is more confusing than it needs to be. The concept here is that coverage of reads at a site cannot include the regions where reads are split and have no sequence. Why use the metric proportion of N-cigar-reads instead of calculating the "sequence coverage at a site" which is coverage/proportion(N-cigar-reads)?
We agree with the reviewer that the concept of coverage and proportion of “N-cigar” reads at a variant site should have been more clearly communicated. Accordingly, we added a new Figure (Fig. 1C) and a clarified description in the third paragraph of the results.

Is the ability to detect variants any different if there are 10 reads covering a site with 0 proportion of N-cigar-reads or if there are 20 reads and 0.5 N-cigar-reads so that the actual coverage of a site is 10?
Yes, indeed. Variant calling from a site with 20 reads and 50% N-cigar reads (i.e., 10 exonic reads and 10 intronic “reads”) represents a more difficult detection problem than 10 reads and 0% N-cigar reads (i.e., 10 exonic reads, and no other overlapping intronic “reads”). For the former site, we would expect lower performance, unless the SplitNCigarReads+flagCorrection steps are performed. We have now added a concrete example in Fig. 1C and a direct comment about the negative effect of intron-containing reads.

Why not have a plot that shows coverage calculated with no flagCorrection vs coverage with flagCorrection and look at the precision and recall in this context. Figure S3 is related but overall uninformative and Figure S4 is confusing because if N-cigar-proportion = 1 then there is no coverage on the variant.

N-cigar reads do not count for read coverage at a site. Thus, the coverage with/without SNCR+flagCorrection would be the same. SNCR+flagCorrection simply splits one long read (composed of exons and introns) into many distinct reads, each containing a single exon. It is possible that a dataset shows N-cigar-proportion = 1 for a site. However, variant calling would not be possible due to lack of exonic coverage. We filter out these sites (i.e., genetic variants in intronic regions) from consideration before benchmarking.
For posterity, we have commented in the text: ”We note also the high proportion of intron-containing reads occurs mostly frequently in lower-coverage regions (Fig. S3), as intron-containing reads do not count for read coverage at a site.” and in the Fig. S3 legend: “N-cigar reads, which represent introns, are not counted towards the total Iso-Seq read coverage quantification of any given site (y-axis).”

Another concern I had was that I felt the scope was a little too narrow and therefore some aspects of analysis were not addressed and so left me with lingering questions about doing this analysis.
What effect does the quality of the initial alignment by minimap2 have on the variant calls? How much would a better alignment matter here?
We recognize that variant calling performance is very dependent on alignment quality. However, alignment quality is also largely determined by the error rates of the original long-read data (and perhaps to a lesser extent on the completeness of the reference genome). There is other recent research that compares how mappability and algorithms used for analysis can impact variant calling (e.g., https://genomebiology.biomedcentral.com/articles/10.1186/s13059-021-02443-7, https://www.sciencedirect.com/science/article/pii/S2666979X2200057X, https://www.biorxiv.org/content/10.1101/2021.07.09.451840v2) and overall, we feel that a serious comparison of alignment quality is beyond the scope of our short report. Moreover, we use a standard aligner, minimap2, which is recommended for Iso-Seq data.

The authors mention in the conclusions that the pipelines should work for Oxford nanopore sequencing and I think the scope would be increased substantially if this data was also included in the investigations of tool performance. There are plenty of publicly available cell line data sets that could be used for this.
PacBio Iso-Seq was the main motivation of our short report, but we agree that comparisons to Oxford Nanopore RNA-seq data are quite interesting and we have added these analyses to the 8th paragraph of the Results (see also Supplemental Figure S8), where we analyzed Nanopore raw subreads (on WTC-11 cells) downloaded from ENCODE, identifier ENCFF961HLO (https://www.encodeproject.org/experiments/ENCSR539ZXJ/).

Similarly, why not use well validated calls from genomic sequencing for validations as well as the complementary short read as tested already? For example, sequencing of Genome in the Bottle sample. Clearly it depends on transcript coverage but so does the RNA-seq data shown and then the coverage for truth data could be determine by the long-read data only. Are there any locations where the long read is actually better than the short read (Indels?).
When we were originally looking for ground truth datasets, we had looked at GIAB, but there were not many long-read RNA-seq (e.g., Iso-Seq) datasets available. In the current project, we use Allen Institute variant calls for the WTC-11 datasets and we used Illumina DNA-seq data to determine the ground truth (see Methods); we think these two datasets provide robust ground truth for testing.
Furthermore, short-read RNA-seq was not the primary motivation in our study, because it is not possible to phase variants linked across long transcripts from short reads alone. But indeed, this is an interesting comparison and we have added a new section that compares performance of short-read versus long-read RNA-seq data to the 9th paragraph of Results (see also Supplemental Figure S9). Perhaps not surprisingly, long reads have improved performance to call indels compared to Illumina short reads.

Reviewer #2: Souza et al. benchmarked several variant callers on PacBio RNA-seq data in this manuscript. And they found that transformation of the alignment files (BAM) is critical to achieve better performance of small variant calling. The idea is interesting. I have some concerns that the authors might consider.
1. It is unclear whether the noisy long-read data has the advantage in detecting variants of RNAs compared with the short-read data. That is the key question of the work and we need a justification to do this work. The authors should compare the performance of variant calling between the short-read data of RNAs and the long-read data. What's the advantage of using long-read RNA data to call variants. As a benchmark paper, many important results were not presented in the manuscript, making it difficult to clearly understand the benchmark result or exact improvement of the performance of the variant calling.
We should clarify (and have now made modifications in the text to make this more clear) that the main purpose of this work is not to compare variant calling between long and short reads, but rather to call variants from long-read RNA sequencing and achieve satisfactory performance. Today, the accuracy of Iso-Seq reads is similar to Illumina RNA-seq. However, the key advantage of using long reads (full-length transcript) is that called variants (from the same gene) can be phased and linked back to isoforms. Moreover, we can better trace the relationship between variants and the encoded protein products.

In the Results, we make the statement: “Although variants called from short reads cannot be phased, which was our original motivation to study variant calling on lrRNA-seq data, we next call variants from Illumina short-read RNA-seq for comparison.”

2. It is not clear how to split the reads at intronic regions. We note that the mode of "splice" of minimap2 was used in the code submitted to github (https://github.com/vladimirsouza/lrRNAseqVariantCalling). Is this related to this purpose?
Whether does a user need a gene annotation file, such as the gff (gtf) file of GRCh38. What to do if the isoform of RNA was not recorded in the annotation file? The authors need to describe how to split the reads in detail in the Methods because the accuracy of read alignment has a big impact on variant calling.
The splitting of reads at intronic regions is simply an operation that GATK’s SplitNCigarReads function performs on BAM files (i.e., given the alignments) to remove the intronic regions. But indeed, the user needs to get good alignments in the first place and minimap2 represents the industry standard. As mentioned above, it is beyond the scope here to also compare alignment algorithms.
As described in the Methods and code, the “splice” mode of minimap2 is used to align long-read RNA-seq to get spliced alignments, which is not directly related to the use of SplitNCigarReads. The use of a reference annotation to guide alignments with minimap2 is up to the user, with argument “--junc-bed” (we do not use it in our analyses).

3. The accuracy of variants depends largely on the read depth, especially for the noisy long-read data. The authors should evaluate the impact of the read depth on the accuracy of the variant calling. For instance, the performance of the variant calling for the total dataset with given median coverage, and for the subsets of different coverages coupled with the site sizes in this dataset. In the end, the authors should give a recommendation on the need of sequencing depth to achieve better performance of variant calling.
Figure 1D and several of the Supplementary Figures show this relationship of performance with respect to read depth. More specifically, we presented variant calling performance from Iso-seq (and now Nanopore RNA-seq in the Supplement, Fig. S8) according to different minimum read coverage thresholds. However, since different sequencing technologies present different coverage features, we realized that subsetting by a minimum coverage may not be a useful comparison between these technologies. To make them more comparable, we now show performance by “coverage ranges” from each sequencing technology in the revision.

4. The authors found that SNCR+flagCorrection+Clair3 had relatively poor performance to call SNPs for Clair3. And the NanoCaller failed to call variants if adding "flagCorrection". The authors claimed that the SNCR+flagCorrection could improve the performance of variant calling

for GATK. However, we did not observe obvious improvement of the calling performances, at least for two tools. This seemed not to match the title of the manuscript "Transformation of alignment files improves performance ...".
Just to clarify, we did not claim that “SNCR+flagCorrection could improve the performance of variant calling for GATK”; in fact, we did not combine flagCorrection with GATK, because GATK is unaffected by this change. We understand the sentiment, but we will stay with the current title because it is well explained in the manuscript to which cases the transformation should be applied. In general terms, the title reflects that these transformations affect results for some tools and in this case, for the most performant tools.

5. Many important definition should be well-defined. For example, In "when read coverage is low...when the coverage is high...", "low" and "high" should be clear and give values or at least a range. The same to the line 45-46 of the page 7: "most of the true variants were called".
Thank you for this comment. Indeed, these statements have now been made more specific in the new text in the revision.

6. The authors claimed that the NanoCaller failed to call variants from Iso-Seq data. The authors of NanoCaller claimed that the F1-score for HG002 CLR data was at least 0.93 for calling SNP.
We suggested that the authors found the reason.
We do not know the nature of exactly why NanoCaller failed on Iso-Seq, but we believe the main difference is simply due the presence of intron-containing reads. The quoted results (F1=0.93 for HG002 CLR) are with respect to DNA sequencing data, but our analysis uses RNA sequencing data. So, we’d expect NanoCaller to perform well in regions distant from a intron-containing reads (e.g., long exons), but the performance was low enough to exclude it from further analyses.

7. Fig. 1D, why did precision decrease as the coverage increased for calling indels using SNCR+GATK? The authors should show the change of variant number for different read coverages.
Using Integrative Genomics Viewer (IGV), we checked several Iso-Seq read alignments around false positive (FP) indels called by SNCR+GATK. In the vast majority of them, we can see both deletion and insertion errors piling up at homopolymers when read coverage is high (see example below). Since GATK's model was developed based on short reads, it does not address

the higher PacBio indel error rate, contributing at least in part to the higher number of FPs.

The change of variant numbers according to read coverage are now shown in supplementary tables (Table S2-S4).

8. Fig. 1F and Fig. 2D, the thresholds of coverage were 30 and 20, respectively. Did the authors have criteria for selecting thresholds?
There were not specific criteria selecting these thresholds, other than to remove lowly expressed regions of the genome.To harmonize the thresholds used in each analysis, we changed the minimum threshold for all UpSet plots from 30 to 20, which is the same used for the splice junction proximity, homopolymer, and ASE analysis.

Reviewer #3: The authors show that variant calling from long read RNA-seq can generally be improved across callers when two different post-processing steps are performed on the bam: splitting reads at introns and correcting flags. They demonstrate this on 2 cell lines relative to a custom "ground truth" from illumina WGS. I expect this will be valuable for the community, but it would likely be good to add a comparison to GIAB benchmark variants, since iso-seq data are now available as described below.
1. GIAB recently made available iso-seq for its HG002 lymphoblastoid cell line (https://www.ncbi.nlm.nih.gov/sra/SRX14226557[accn], which also has illumina RNA-seq at https://www.ncbi.nlm.nih.gov/sra/SRX12200334[accn]) as well as two different iPSC lines from the same HG002 individual that have more genes expressed (https://www.ncbi.nlm.nih.gov/sra/SRX14226558[accn] and https://www.ncbi.nlm.nih.gov/sra/SRX14226556[accn]). GIAB plans to make additional short and long read RNAseq data available shortly. Importantly, variants from these could be compared to the latest GIAB benchmarks: v4.2.1 vcf and bed (which used short, linked, and long reads for the whole genome at https://doi.org/10.1016/j.xgen.2022.100128), and the 273 challenging medically relevant gene benchmark vcf and bed at https://rdcu.be/cGwVA. These benchmark variants and regions would likely provide more accurate performance metrics, since they used multiple technologies and were highly evaluated.
As mentioned above, when we were originally looking for ground truth datasets, we had looked at GIAB, but there were not many long-read RNA-seq (e.g., Iso-Seq) datasets available at the time. We agree that the data available there provides great test cases, we also think that the two datasets we used provide robust ground truth for testing and feel that it is out of scope at this stage.

2. What are the n=x counts in Fig 2C? I'm surprised n is so large for mid-size homopolymers
It is now stated in the legend of Fig. 2 that “n indicates the number of true variants covered by Iso-Seq data for each calculated performance measure”.

3. In Fig 2D, I'd expect the effect of ASE to depend on both how strong the ASE is and what coverage the ASE site has in PacBio. Would the authors be able to look at either of these factors as well?
4. It also seems like it could be useful to look at the effect of coverage on variant detection at non-ASE sites
These both indeed could be interesting analyses, but our main intention with the ASE analyses is to highlight that there are (obvious) blind spots when using RNA-seq data for genotyping. If the editor feels these are crucial elements to add, we would be happy to do them. But in keeping with the spirit of a short report, we have already demonstrated the main bias that ASE imposes on variant calling.

**2^nd^ round**

**Reviewer 1**

There is still some confusion for me around the purpose of this paper. The title indicates it's proposing a correction tool to improve variant detection and the abstract says the paper benchmarks variant calling tools. However, I feel that neither is fully achieved and leads to confusion especially in the introduction. For a good benchmarking it would be good to see some simulations to really understand the relationships in the observations of accuracy, coverage, intronic coverage, ASE, spice junctions etc. In particular the ASE study is really cursory. However I actually question if this really is a benchmarking study in the same sense as many of the other papers from this lab.

The addition of figure 1C could be useful if that actual wording was consistent throughout the paper. "Coverage" isn't referred to in 1C but you use coverage everywhere like 1D and the reader has to assume it's exonic reads from 1C but it's not clear. See these sentences for example:
"In such a case, the presence of intron-containing reads close to the variant can "spoil" variant calling accuracy"
"We note also the high proportion of intron-containing reads occurs more frequently in lower-coverage regions (Fig. S3; Fig. 1C, sites S2 and S4), as intron-containing reads do not count for read coverage at a site. Taken together, this explains why DeepVariant alone showed a closer performance to SNCR+flagCorrection+DeepVariant when coverage is high (e.g., 80-100; see also Fig. S2), which typically involve cases of exonic regions and hence,
SNCR+flagCorrection make little difference"

What does "close to" mean? I assume "spoil" means reduce? How do intronic reads make a difference apart from coverage (I asked this in my last review but I still don't understand the evidence). I assume that DeepVariant just uses the total read coverage, regardless of if it's an intron or exon, to call variants unless you manipulate the alignments. Why not just say that. The way it's currently communicated is so convoluted. You show that accuracy increases with coverage (which doesn't include intronic reads) and decreases with the proportion of intronic reads that you try to control for coverage. The read manipulation is just making sure each base get the correct coverage calculated if I understand correctly.

I don't like the use of "DeepVariant alone". Please refer to it as DeepVariant with no alignment manipulation or something similar.

The initial "motivation" for variant calling on long reads is phasing and yet there is not mention of this in the results or even if the tools are capable of this or what it takes to get phased variants.

Minor
Why do the read coverage ranges not include all coverages, e.g. table S2?

**Reviewer 2**

The authors have addressed all my concerns and I do not have further concerns.
